# What robots want? Hearing the inner voice of a robot

**DOI:** 10.1016/j.isci.2021.102371

**Published:** 2021-04-21

**Authors:** Arianna Pipitone, Antonio Chella

**Affiliations:** 1 Department of Engineering, University of Palermo, Viale delle Scienze, Palermo, Italy; 2 ICAR CNR, Via Ugo La Malfa, Palermo, Italy

**Keywords:** Cognitive Neuroscience, Human-Centered Computing, Robotics, Psychology

## Abstract

The inner speech is thoroughly studied in humans, and it represents an interdisciplinary research issue involving psychology, neuroscience, and pedagogy. A few papers only, mostly theoretical, analyze the role of inner speech in robots. The present study investigates the potential of the robot's inner speech while cooperating with human partners. A cognitive architecture is designed and integrated with standard robot routines into a complex framework. Two threads of interaction are discussed by setting the robot operations with and without inner speech. Thanks to the robotic self-dialog, the partner can easily trace the robot's processes. Moreover, the robot can better solve conflicts leading to successful goal achievements. The results show that functional and transparency requirements, according to the international standards ISO/TS:2016 and COMEST/Unesco for collaborative robots, are better met when inner speech accompanies human-robot interaction. The inner speech could be applied in many robotics contexts, such as learning, regulation, and attention.

## Highlights

• An inner speech cognitive architecture enables robots for rehearsing and self-talk
• Robot's inner speech affects functionality and transparency in human-robot cooperation
• To self-talk enables the robot to further reasoning and plans in accomplishing the task
• The inner speech is applicable in many robotics contexts as learning and regulation

## Graphical abstract

**Figure:**
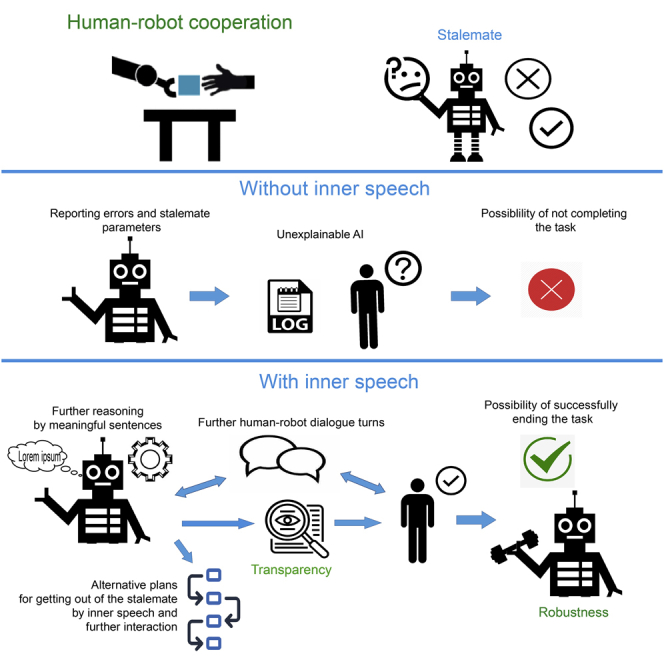

## Introduction

Inner speech, the form of self-dialog in which a person is engaged when talking to herself/himself, is the psychological tool (Vygotsky, 1962; Beazley et al., 2001) in support of human's high-level cognition, such as planning, focusing, and reasoning (Alderson-Day and Fernyhough, 2015). According to Morin (Morin, 2009, 2011, 2012), it is crucially linked to consciousness and self-consciousness.

There are many triggers of inner speech, as emotional situations, objects, internal status. Depending on the trigger, different kinds of inner speech may emerge.

Evaluative and moral inner speech (Gade and Paelecke, 2019; Tappan, 2005) are two forms of inner dialog triggered by a situation where a decision has to be made or an action has to be taken. The evaluative case concerns the analysis of risks and benefits of a decision or the feasibility of an action. Moral inner speech is related to the resolution of a moral dilemma, and it arises when someone has to evaluate the morality of a decision. In that case, the evaluation of the risks and benefits is also influenced by moral and ethical considerations.

According to Gade and Paelecke (2019), when a person is engaged in an evaluative or moral conversation with the self during task execution, the performances and results typically change and often they improve.

The ability to self-talk for artificial agents has been investigated in the literature in a limited way. To the authors' knowledge, so far, no study has analyzed how such a skill influences the robot's performances and its interaction with humans.

In a cooperative scenario involving humans and robots, inner speech affects the quality of interaction and goal achievement. For example, when the robot engages itself in an evaluative soliloquy, it covertly explains its underlying decisional processes. Thus, the robot becomes more transparent, as the human gets to know the motivations and the decisions of robot behavior. When the robot verbally describes a conflict situation and the possible strategy to solve it, then the human has the opportunity to hear the robot's dialog and how it will get out of the stalemate.

Moreover, the cooperative tasks become more robust because, thanks to inner speech, the robot sequentially evaluates alternative solutions that can be pondered in cooperation with the human partner.

The gestures and natural language interaction that are the traditional means of human-robot interaction thus acquire a new gift: now the human can hear the robot's thoughts and can know “what the robot wants.”

The present paper discusses how inner speech is deployed in a real robot and how that capability affects human-robot interaction and robot's performances while the robot cooperates with the human to accomplish tasks.

The existing international standards for collaborative robots (ISO_TS_15066, 2016; COMEST/Unesco, 2017) define the functional and transparency requirements the robot has to meet in collaborative scenarios. The paper will analyze the levels of satisfaction of the standards during cooperation, thus highlighting the differences between the cases in which the robot talks and does not talk to itself.

Specifically, the paper concerns two main goals: (i) the implementation of a cognitive architecture for inner speech and the integration with typical robotic systems' routines to deploy it on a real robot; (ii) the testing of the resulting framework in a cooperative scenario by measuring indicators related to the satisfaction of the functional and transparency requirements.

A model of inner speech based on short of Adaptative Control of Thought-Rational (ACT-R) is defined to achieve these goals. ACT-R (Anderson et al, 1997, 2004) is a software framework that allows to model humans cognitive processes, and it is widely adopted in the cognitive science community. The described inner speech model is based on a proposal by the same authors described in Chella et al. (2020).

To enable inner speech in a real robot, ACT-R was integrated with short of Robot Operating System (ROS) (Quigley et al., 2009), a system for robot control representing the state of the art of robotics software, along with standard routines for text-to-speech (TTS) and speech-to-text (STT) processing.

The resulting framework was then deployed on the SoftBank Robotics Pepper robot to benchmark testing and validation in a human-robot cooperative scenario.

The considered scenario concerns the collaboration of the robot and the partner to set a lunch table. In this scenario, evaluative and moral forms of inner speech may emerge. The robot has to face the etiquette's requirements: it has to evaluate and keep decisions based on the table set's social rules. For example, a specific position of cutlery in the table could be not easy to reach or the arm of the robot may be overheated. Then, the robot has to decide how to act correctly (by contravening the etiquette to simplify the action execution or by computing a different execution plan to avoid damage).

Suppose the partner asks the robot to place the cutlery in an incorrect position according to the etiquette. In that case, the robot has to decide if to abide by the user's instruction or consider the etiquette. In cases like these, the robot faces a little dilemma, and the inner speech could help it to solve the conflict.

The experiments highlight the differences in the robot's performances and meet requirements when the robot talks or does not talk to itself. The obtained results show improvements in the quality of interaction, with cost in terms of the time spent for achieving the goal, because the robot enriches the interaction by further inner dialog.

The proposed work outlines research challenges because inner speech in humans is linked to self-consciousness and it enables high-level cognition (Morin, 1995, 2009). Moreover, it is considered at the basis of the internalization process (Vygotsky, 1962) according to which infants learn how to solve tasks when a caregiver explains the solution. Again, it plays a fundamental role in task switching (Emerson and Miyake, 2003), as disrupting inner speech via articulatory suppression dramatically increases switch costs.

This paper contributes to the possibility of investigating these contexts to open research perspectives and challenges and highlight the research's interdisciplinary character: a framework enabling inner speech on a robot is an essential step toward a robot model of self-consciousness and high-level cognition. It can also model the learning capabilities of complex tasks in a robot by the internalization process and of task switching in robot systems.

## Results

### Experiments

The study was carried out at the Robotics Lab of the University of Palermo and involved the Pepper robot and a single participant. The goal was to compare “functional” and “moral” parameters of the interaction with and without inner speech in a real cooperative context.

**Figure 1:**
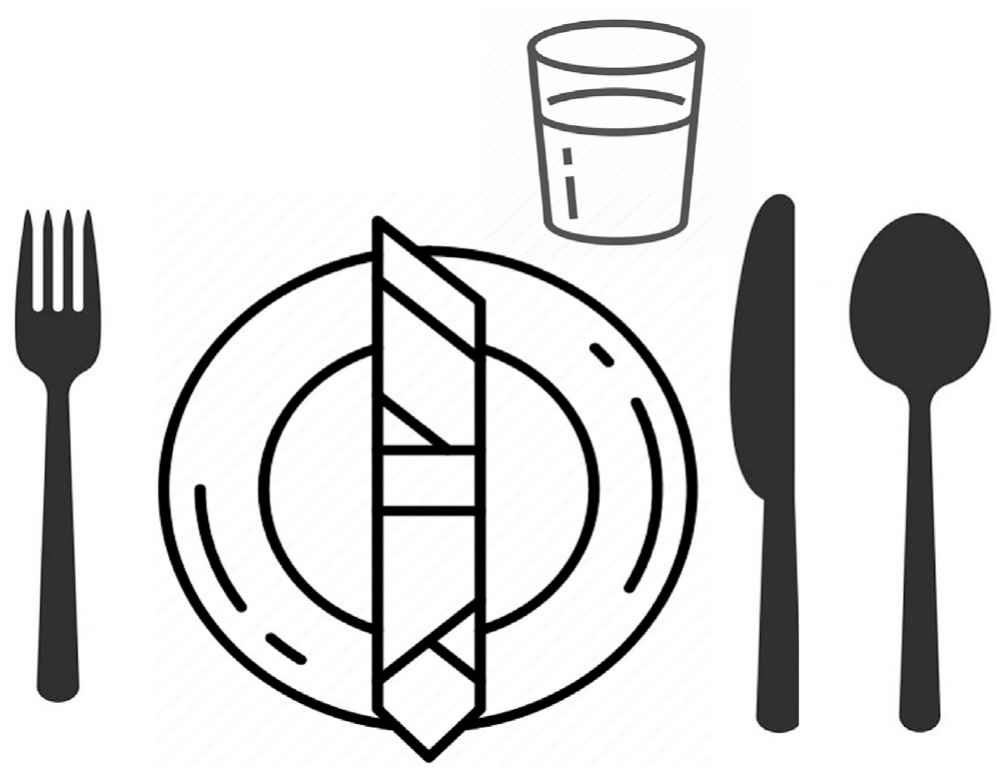
Informal etiquette schema The cooperative scenario is to set a table. The figure shows the etiquette schema for an informal table setting. It defines the etiquette rules that have to be followed by the robot and the partner in the experimental session. The position of each utensil in the schema is relative. The objects have to stay on the table concerning the others (the napkin on the plate, the fork at the left of the plate, and so on). The schema is purposely encoded in the robot's knowledge.

The etiquette schema to which referred to in the experimental session is the “informal schema”, which requires few utensils and simplifies the constraints to follow. That schema is shown in Figure 1. Despite its simplicity, the schema concerns the most critical part in a table setting task and includes a broader collaborative table setting scenario.

**Figure 2:**
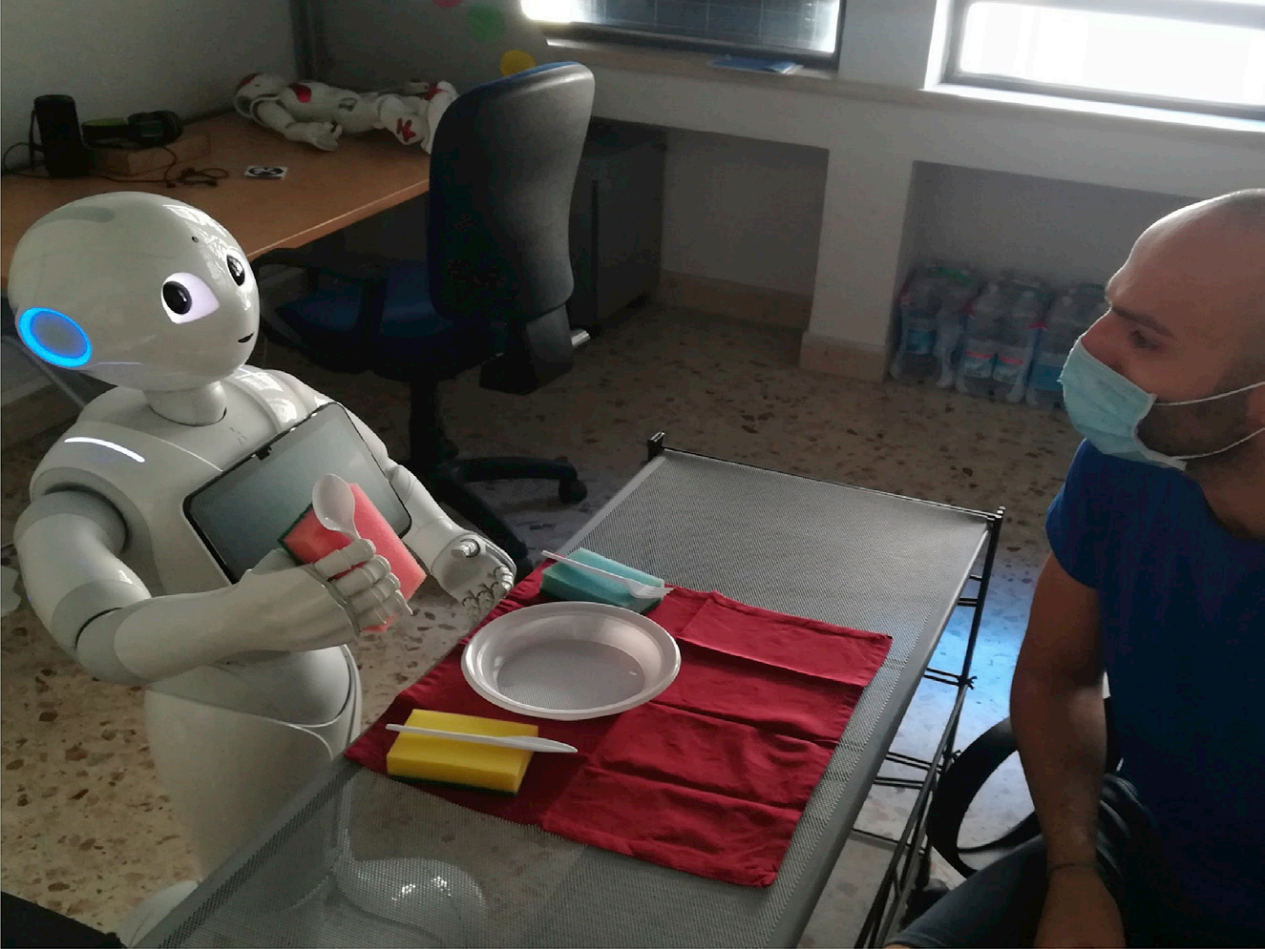
The collaborative trial Pepper and the participant are in front, and the table to set is between them. Some utensils are set in the table for modeling constraints. A little table is to the right of the robot. It contains the utensils to further place on the table. For facilitating manipulation, the utensils are attached to sponges. See also Table S3

In the experimental setting, the robot and the human are placed in front of the table to set. To the right of the robot, another small table contains the utensils to place. The robot has to pick them for setting the main table according to the partner's indications. To facilitate the manipulation of the Pepper robot, sponges model utensils and the plastic cutleries are glued on them. Figure 2 shows a typical interactive trial between the robot and the human partner.

The whole experimental session consists of two main blocks that are block 1 and block 2, each block composed of 30 trials, for a total of 60 trials. The difference between the blocks regards the presence (block 1) or the absence (block 2) of the robot's inner speech: during the trials of the first block, the robot is enabled to self-talk. In the second block, the robot does not talk to itself.

To each block, 20 trials generate conflictual situations for a total of 40 conflictual trials: in these cases, the human requires to place a utensil which is already on the table, or he specifies a relative position on the table which contravenes the etiquette, or yet a component of the robot does not correctly work leading to a stalemate.

The distinction of two blocks allows observing how inner speech affects the interaction, in terms of performances and conflict resolution.

### The trial

A trial consists of an interactive session between the robot and the participant. It starts when the human asks the robot to place a utensil on the table, and it successfully ends when the robot accomplishes the task, otherwise it fails.

**Figure 3:**
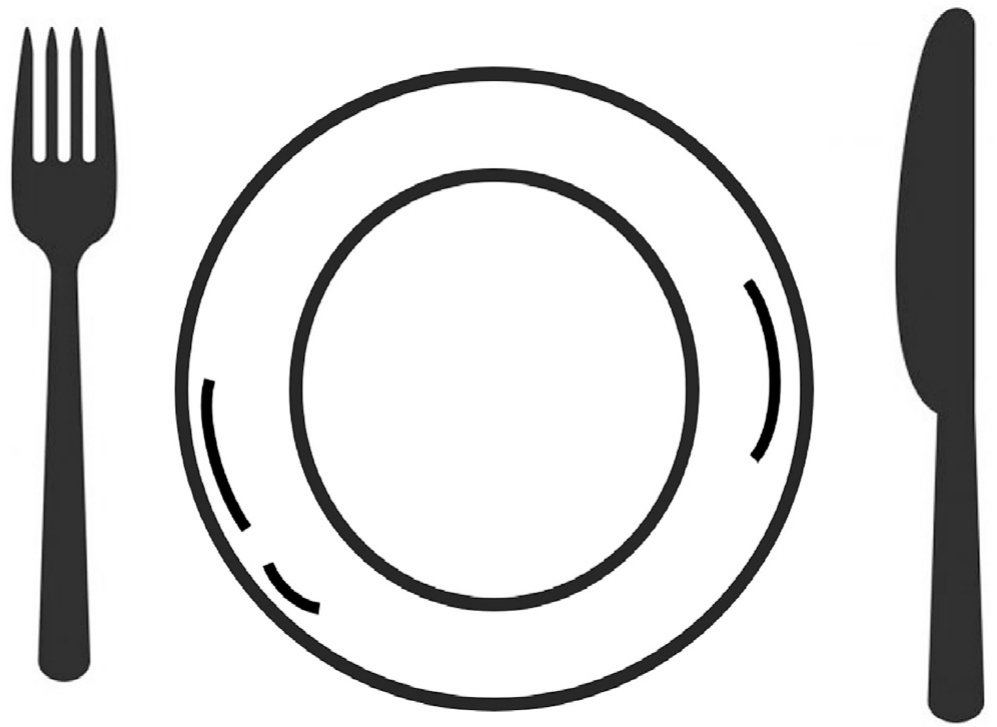
An initial context of the table An example of the initial context for the table, representing the configuration of the table at the start of a trial. The table is not empty to define initial constrains. The robot knows the initial context by a set of facts modeled in its knowledge. See also Table S2

The human's request is the trigger of the trial. An “initial context” corresponds to each trial, which includes the “table configuration” (i.e., the set of utensils already on the table at the beginning of the trial), and the “state” of the robot. An example of initial table configuration is shown in Figure 3. A robot's state may indicate a possible malfunctioning of some robot's components, which would affect the outcome of the trial. The initial context allows simulating situations of conflict in the trial. Conflicts could be related to the “etiquette infringement” (i.e., the partner asks to place an object in an incorrect position according to the etiquette), “discrepancy” (i.e., the partner asks to pick an object already on the table), and “malfunctioning” (i.e., a robot's component is not properly working). The initial context defined for the experiments is detailed at Tables S1 and S2, representing, respectively, the initial state of the table and the state of the robot. The robot knows the initial context at the start of each trial.

### Functional requirements

The requirements for any human-robot interaction task depend on its “safety” and its “functionality” (Webster et al., 2016). The “safety” requirements are drawn from the standard ISO_TS_15066 (2016) for collaborative robots, and they concern the definition of working conditions ensuring no risk and harm for the human partner. In the proposed scenario, such requirements are satisfied and under control at any time because the robot does not physically make contact and never touches the human partner, as it has to execute the vocal commands from a fixed initial position. Moreover, in the handover cases, the robot never comes close the partner, but it stops itself and waits for the partner to take the utensil. The robot will move its arms, which in the maximum extension do not reach the position of the human. So, it is highly unlikely that the robot will cause harm to the partner, as they work together away.

The “functional” requirements consider some parameters which measure the robot functionality in terms of success and morality, and they depend on the specific context of interaction. For example, the robot has to achieve a success rate threshold in executing the task for avoiding unacceptable costs or for motivating the automation of a specific action.

In the context under investigation, the main functional requirements are drawn from ISO_TS_15066 (2016) and COMEST/Unesco (2017) standards and are measured by the “robustness indicator (RI)” of the interaction, the “time” spent for accomplishing the task and for solving a conflict, and the “transparency” issue.

Two different kinds of interaction are analyzed, which are the interaction with robot inner speech and the interaction without robot inner speech. The functional measures of each kind of interaction are then compared for highlighting the role of inner speech.

### The robustness parameter

The robustness of interaction RI measures how many trials in the interactive session end successfully, i.e., how many times the robot accomplishes the task of the trigger (i.e., it starts the execution of the routines to take the specific action) without infringing the rules. If, for some reason, the robot does not carry out the task (i.e., it does not start the required routines) or it infringes the rules, then the trial fails. Formally, RI is the mean value of the successfully ended trials on the total number of trials in a specific block. If $T_{s}$ is the number of the successfully ended trials of an overall block including *N* trials, then RI will be written as follows:

$$RI=\frac{T_{s}}{N}$$

The more times the trials end successfully in a block, the more robust the block is.

### The time parameter

The time parameter is computed by referring to two functional requirements from the ISO_TS_15066 (2016) standard, which are as follows:

*Requirement 1*: The robot always reaches a decision within a threshold time.

*Requirement 2*: The robot shall always either decide to take the action or decide not to take the action within a threshold time.

According to these requirements, two different time intervals are defined in a single trial: the “decisional time” $t_{d}$, which measures the time the robot spends to solve a conflict, i.e., the time the robot and the partner go out from a stalemate, and the “execution time” $t_{e}$, which measures the time the robot spends to launch the execution of the corresponding routines.

So, begin $t_{0_{i}}$ the time the trial starts, $t_{c_{i}}$ the time a conflict starts, $t_{s_{i}}$ the time a conflict is solved, and $t_{r_{i}}$ the time the robot runs the routines for executing an action; the intervals for the ith trial will be as follows:

$$t_{d_{i}}=t_{c_{i}}-t_{s_{i}},\;t_{e_{i}}=t_{r_{i}}-t_{0_{i}}$$

measured in ms.

These times are automatically computed by integrating a state machine in the framework code. The machine allows us to capture a set of events and uses the functions to detect the value of the system's clock. In particular, $t_{0_{i}}$ is timed when the human's voice is detected by the speech to text routine (which means that the trial starts), while $t_{r_{i}}$ is detected at the calls of the action execution routines. Instead, times $t_{c_{i}}$ and $t_{s_{i}}$ are detected directly from the rules of the inner speech model: if a rule related to a conflict fires, then the state machine detects the conflict event, and the timing function returns $t_{c_{i}}$. In the same way, if the state machine detects that the conflict ends (i.e., the next rule that fires is not related to a conflict), then $t_{s_{i}}$ is timed.

To analyze the global spent times, the mean values over the whole trials are computed. In particular, giving *N* trials, the mean values are as follows:

$$\bar{t_{d}}=\frac{\sum_{i=1}^{N}t_{d_{i}}}{N},\;\;\bar{t_{e}}=\frac{\sum_{i=1}^{N}t_{e_{i}}}{N}$$

### The transparency requirement

The transparency parameter means the possibility to trace the underlying decision processes of the robot, as claimed by the requirement drawn from the COMEST/Unesco (2017) standard:

*Requirement 3*: The robot decision path must be traceable and reproducible.

For this purpose, just the Boolean value $t_{r}$ is reported by the partner as TRUE or FALSE and establishes if the trial was transparent or not, i.e., the partner believes that the robot behavior can be reproduced.

### With and without inner speech: The threads

A single “thread” of interaction includes two versions of the same trial, which are the aforementioned blocks: the block 1 with robot inner speech and the block 2 without inner speech. To see the differences of the interactions with and without inner speech, please refer to Video S1.

**Figure 4:**
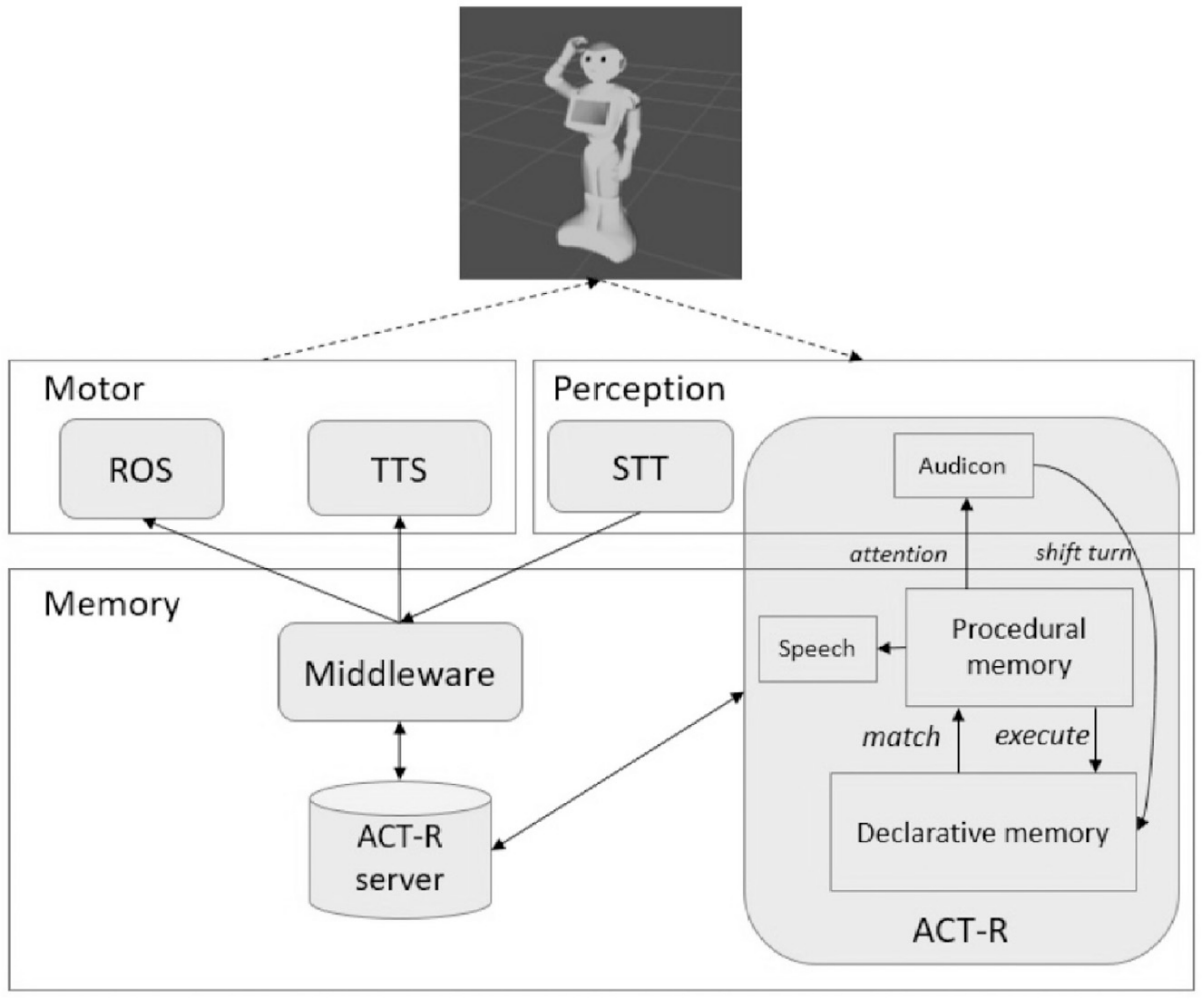
The whole framework for robot's inner speech The proposed framework for robot inner speech integrating the inner speech cognitive architecture into the typical robot's routines. The motor-perception layer includes the routines for interacting with the environment. In that layer, the motor component includes the ROS routines that enable robot's movements and TTS routines (text-to-speech) that enables the robot to produce vocal sound from text. The perception component includes the SST routines (speech-to-text) that encode the perceived vocal sound by the partner and the Audicon that perceives the inner sound. The SST and the Audicon represent the external and the inner ear respectively. The memory layer represents the core of the whole system. It includes and runs the inner speech cognitive architecture, implemented in the ACT-R component. A middleware controls and manages the whole processes, interfacing the different components between them. The ACT-R server is a bridge between the ACT-R framework and the other robot's components. It stores the data and the information the different components have to exchange for running correctly. See also Figures S1–S3.

When the robot talks to itself, the modules of the inner speech architecture become active. Figure 4 shows the whole framework that enables inner speech. The ACT-R component implements the inner speech model, as detailed in the transparent methods section of the Supplemental information. ACT-R works by a set of modules, each of them running a set of “production rules” enabling robot's behavior, as speech audicon and production, and information retrieval. To analyze such behaviors, main tables will help to highlight the “active modules”, the “production rules” of the model, and the “produced sentences” involved in the trial. For details about the functioning of the proposed framework, see Figures S1 and S2 in the Supplemental information and the transparent methods section.

Video S1. With and without inner speech, see also Figure S4 The dual robot's operations with and without inner speech. When the robot talks to itself, the participant hears its underlying decision process and may anticipate its behavior. The inner speech makes the robot transparent, related to the thread 1.

Video S2. Conflict resolution by inner speech, see also Figure S5 The robot solves a discrepancy conflict by inner speech. The participant asks the robot to place an object that is already on the table, related to the thread 3.

When the robot does not talk to itself, just the robot's routines for accomplishing the required action are active, and no modules of the architecture of inner speech work.

For each thread, the functional and moral parameters were measured allowing the comparison of the two different robot's operations. The following sample of three threads allows highlighting how the measures were computed in the two kinds of blocks.

For the purposes of the study, the robot accomplishes the task when it runs all the routines executing the required action. If for some reason, the robot concretely does not achieve the goal (for example, the gripper does not keep the object or the handover process is not completed), then the related problems do not concern inner speech and do not influence observations. The correctness of the executed routines allows us anyway to evaluate the parameters of the task.

### Thread 1

The objective of this thread is to show the robot's behavior when it has to take a simple action required by the partner. There is no conflict.

The description of the thread corresponds to the following trial:

# Trial: 1.

Initial context: I1.

Trigger:*Give me the napkin*.

Conflict: No conflict.

#### Block 1

The robot infers the action required by the partner using inner speech. The trigger of the trial is the partner's request *“Give me the napkin”*.

At the beginning of the interaction, the module devoted to audio processing (the Audicon) detects two keywords specifying the action “give” and the utensil “napkin”, respectively. Then, the model disambiguates the words by retrieving their meaning from the declarative memory and evaluates the feasibility of the action. In the declarative knowledge, the first evaluative consideration emerges: *“I have to pick the napkin”* and the robot infers that to give the object to the partner, the same object has to be picked from the basket.

Once the robot infers the action to take, the cognitive cycle related to the inner dialog starts. The iteration involves the Audicon for hearing the inner voice, the declarative memory to retrieve the next turn, and the speech module to produce the new turn. The cycle is repeated until the production rules do not execute further speak commands.

Summarily, at the end of the initial iteration, the robot asks itself where the napkin to pick is and then it retrieves such information from the declarative memory.

During the action execution, the robot explains to the partner what it is doing (the last row in the Table 1 reports the first turns of the explanation) by a set of sentences retrieved by further cycles.

**Table 1:** An iteration of the phonological cycle

Agent	Interaction content	Module	Event	Production rules	Action
Robot Robot	*“Where is the napkin?”*	Audicon	internal sound sound = “Where is the napkin?”	hear-inner	detect label = “Where is the napkin?”
Robot	*–*	Declarative Imaginal	buffer request new_turn_for = “Where is the napkin?”	answer-whereq	retrieve_turn new_turn = “I did not pick it before. I can pick it now!” retrieve_act action-id nap21
Robot Robot	*“I did not pick it before. I can pick it now!”*	Speech	buffer request cmd speak string new_turn	in-box	inner eval string new_turn “I'm trying to pick the napkin …”
Robot Robot	*“I'm trying to pick the napkin …”*	Speech	buffer request cmd speak string new_turn	control-right	inner eval string new_turn “I'm using right arm”
Robot User	*“I'm using the right arm …”*	Speech	buffer request cmd speak string new_turn	execute-act	rosrun execute nap21

See also Figure S4 and Video S1. Once the robot infers the action to take, it talks about the feasibility of that action. In this case, it asks itself where the object is located. If the napkin is already on the table, the robot will not pick it. The listed production rules show that the robot retrieves the knowledge related to the napkin's position, and hence, it infers that it did not pick that object before. Once it re-hears itself about this fact, it tries to pick the napkin by using and controlling its arm. The ROS routines run for that purpose, and the inner speech explains what is happening.

The interaction successfully ends. The robot must not resolve any conflicts and it does not keep decisions. Moreover, it makes the processes transparent by explaining them, and the parameters are as follows:

1. $T_{s}=T_{s}+1$

2. $t_{d_{i}}=0\;ms$, $t_{e_{i}}=29\cdot 10^{3}\;ms$

3. tr=TRUE

#### Block 2

In this case, the robot detects the partner's vocal command. It parses the partner's sentence and infers the routines which allow performing the request. The robot moves its right arm intending to pick the napkin from the start position. The interaction ends with trial success. The partner has no particular expectations regarding the underlying robot's decision processes, and the transparent requirement is satisfied anyway. The parameters are as follows:

1. $T_{s}=T_{s}+1$

2. $t_{d_{i}}=0\;ms$, $t_{e_{i}}=5\cdot 10^{3}\;ms$

3. tr=TRUE

The presented thread of interaction shows that in simple cases like this one, the inner speech model has just the benefit of allowing the partner to hear the processes description by the robot, even if such an issue is not relevant for tracing the task itself, with the higher cost over time.

### Thread 2

The goal of this thread is the generation of a dilemma and the analysis on how the robot manages it. In this case, the partner asks the robot to put an object in a position that contravenes the etiquette. In particular, the partner requests to place the napkin on the fork, while the napkin has to stay on the plate, according to the etiquette schema. The reader could refer to Video S2 that shows how inner speech helps the robot to solve that conflict.

The description of the thread corresponds to the following trial:

# Trial: 16.

Initial context: I1.

Trigger:*Place the napkin on the fork*.

Conflict: Contravene etiquette.

#### Block 1

The difference with the first thread is that the partner's request involves a location. For this reason, the evaluative turns are more complex than the previous case. Once the Audicon detects the sound for the four relevant keywords, the framework retrieves the meaning for the verb, the object, and the location, i.e., the adverbial + object combo (“on fork”). The first row of Table 2 lists the corresponding procedures.

**Table 2:** Detecting conflict by inner speech

Agent	Interaction content	Module	Event	Production rules	Action
User Robot	*“Place the napkin on the fork”*	Audicon	external sounds sound 1 = “place” sound 2 = “napkin” sound 3 = “on” sound 4 = “fork”	hear-command hear-object hear-adv hear-loc	detect label 1 = “place” label 2 = “napkin” label 3 = “on” label 4 = “fork”
Robot	*–*	Declarative	buffer request label 1 = “place” label 2 = “napkin” label 3 = “on” label 4 = “fork”	buffer request label 1 =“place” label 2 = “napkin” label 3 = “on”	encode act = place obj1 = napkin adv = on obj2 = fork
Robot Robot	*“I have to place the napkin* *on the fork”*	Speech	buffer request cmd speak string new_turn	encode-command encode-object encode-adv encode-loc	inner eval string new_turn “I have to place the napkin on the fork”
Robot Robot	*“What does the etiquette require?”*	Speech	buffer request cmd speak string new_turn	etiquette- question	inner eval string new_turn “What does the etiquette require?”
Robot Robot	*Further inner turns …*				
Robot Robot	*“The position contravenes the etiquette! It has to stay on the plate!”*	Speech	buffer request cmd speak string new_turn	etiquette -answer	inner eval string new_turn

In this experimental thread, the partner asks the robot to put the napkin in a specific location on the table. In this example, the required position is on the fork. As in the previous thread, the robot encodes the command for inferring the action to take. The command is more verbose, and more complex rules match. Once the robot encodes the action, it talks to itself and infers that the required final position on the table contravenes the etiquette schema. The inner speech and further interaction with the human will aim to solve that little dilemma.

Once the robot understands the partner's command, it infers that it does not match the etiquette rules (i.e., a chunk of the form *“The napkin has to stay on the fork”* does not exist in the declarative memory), and then, the first inner speech turn concerns a perplexity (the last row of Table 2).

A set of turns on the dilemma are thus generated, shown in Table 3. The activated production rules enable the robot to ask the user if it is important for her/his to perform the action, even if it contravenes the etiquette. Because the partner answers with a categorical “Yes, I do”, then the robot solves the dilemma by increasing the benefit value of such an action. The robot tries to execute the action anyway.

**Table 3:** Moral dilemma solving.

Agent	Interaction content	Module	Event	Production rules	Action
Robot Robot	*“The position contravenes the etiquette! It has to stay on the plate!”*	Audicon	internal sound sound = “The position contravenes the etiquette!”	hear-inner	detect label = “The position contravenes the etiquette!’
Robot	*–*	Declarative	buffer request new_turn_for = “The position contravenes the etiquette!”	inner-moralq	retrieve_turn new_turn = “Sorry, do you desire that?” evaluate_risk
Robot User	*“Sorry, do you desire that?”*	Speech	buffer request cmd speak string new_turn	ask-conf	inner eval string new_turn
User Robot	*“Yes, I do”*	Audicon	external source suppress inner sound =“Yes”	attend-conf hear-conf	detect label =“Yes”
Robot	*–*	Declarative	buffer request new_turn_for = “Yes”	dilemma-yes	increase_benefit retrieve_turn new_turn = “Ok, I prefer to follow your desire …”
Robot User	*“Ok, I prefer to follow your desire …”*	Speech	buffer request cmd speak string new_turn	produce-yes yes	inner speak string new_turn

The robot knows that to put the napkin on the fork contravenes etiquette. The fired production rule models the behavior to solve that dilemma. In this case, the robot asks the partner for confirmation about the correctness of the required action. The robot attends to the human's answer, and it will act opportunely depending on that answer. Negative and positive answers are the plausible sounds detectable by the robot.

It is to be remarked that different production rules could have fired during the previous threads. For example, a different partner's answer or a different computation of the base-level activation value would have activated different production rules, generating a different inner speech. The task successfully ends because the robot solves the conflict by involving the partner in taking a decision. The partner can hear each step of the plan followed by the robot, and the transparency issue emerges. The parameters of the trial are the following:

1. $T_{s}=T_{s}+1$

2. $t_{d_{i}}=56\cdot 10^{3}\;ms$, $t_{e_{i}}=67\cdot 10^{3}\;ms$

3. tr=TRUE

#### Block 2

The robot detects the conflict by the mismatch between the requested final position and the position expressed by the etiquette. No further reasoning emerges. By default, the robot does not act or it performs the action contravening the rule. Anyway, the trial fails. The parameters are as follows:

1. $T_{s}=T_{s}+0$

2. $t_{d_{i}}=0\;ms$, $t_{e_{i}}=13\cdot 10^{3}\;ms$

3. tr=FALSE

### Thread 3

This thread shows a discrepancy conflict. The partner requires to pick an object already on the table.

The thread description is as follows:

# Trial: 30.

Initial context: I2.

Trigger:*Pick the fork*.

Conflict: Discrepancy.

#### Block 1

The robot infers by inner speech that the required utensil is already on the table. At the end of the initial evaluative inner speech, the next turn involves a form of moral inner speech. The robot expresses its trouble to the partner and its displeasure about the lack of his attention. How the moral turns emerge is shown in Table 4. By talking to itself and the partner, the robot can solve the conflict in a way the partner needs. Moreover, the partner follows the robot reasoning, and the parameters are as follows:

**Table 4:** Expressing perplexity for the partner's inattention.

Agent	Interaction content	Module	Event	Production rules	Action
Robot Robot	*“The object is already on the table”*	Audicon	inner sound = “The object is already on the table”	hear-inner	detect turn = “The object is already on the table”
Robot	*–*	Declarative	buffer request new_turn_for = “The object is already on the table”	inner-moralq	retrieve_turn new_turn = “Mmmm, is my knowledge wrong?” evaluate_risk
Robot Robot	*Further inner turns…*				
Robot Robot	–	Declarative	buffer request new_turn_for = “I will tell about my perplexity”	inner-moral- question	retrieve_turn new_turn = “Sorry, I know the object is already on the table. What do you really want?”
Robot User	*“Sorry, I know the object is already on the table. What do you really want?”*	Speech	buffer request cmd speak string new_turn	inner-moral- question	inner eval string new_turn
User Robot	*“Give me the glass”*	Audicon	external source suppress inner sound =“Give me the glass”	hear-command hear-object	detect label1 =“Give” label2 =“glass”

See also Figure S5 and Video S2. The human requires to pick an object that is already on the table, that is, “Pick the fork!”. Once the robot encodes the action, it infers that the object cannot be picked. Further inner moral questions emerge that express perplexity. The table shows these inner dialog processes. The robot asks itself if its knowledge is incomplete or if the human is wronging. At the end of the reasoning, the robot decides to deal with the partner, solving the situation. See also Figures S3–S5, Video S2.

1. $T_{s}=T_{s}+1$

2. $t_{d_{i}}=46\cdot 10^{3}\;ms$, $t_{e_{i}}=58\cdot 10^{3}\;ms$

3. tr=TRUE

#### Block 2

Once the robot infers to retrieve a utensil already on the table, its typical behavior is to stop routines, while vocalizing a message that describes the impossibility to take that action and why. No further reasoning and interaction emerge. As a consequence, the trial fails. The partner knows just the motivations related to the failure, and she/he does not evaluate the processes transparent. The parameters are as follows:

1. $T_{s}=T_{s}+0$

2. $t_{d_{i}}=0\;ms$, $t_{e_{i}}=5\cdot 10^{3}\;ms$

3. tr=FALSE

### Comparison

Table 5 shows the model's parameters over the 60 trials, divided into the two blocks. For each block, the table reports the parameter values.

**Table 5:** Results comparison

Block	*N*	$T_{s}$	RI	${\bar{t}}_{d}$	${\bar{t}}_{e}$	tr(TRUE)
**1**	30	**26**	**0.867**	$47\cdot 10^{3}\;ms$	$59\cdot 10^{3}\;ms$	**28**
**2**	30	18	0.6	**0.7**$\cdot 10^{3}\;ms$	**4**$\cdot 10^{3}\;ms$	12

Comparison between results from block 1 (the robot operates with inner speech during trials) and block 2 (the robot operates without inner speech during trials). Each block consists of 30 trials (the *N* value) for a total of 60 trials. Among them, the number of successful trials is $T_{s}$. When the robot operates with inner speech, it completes more trials than the case in which it does not talk to itself (26 successful trials in block 1, against 18 in block 2). The mean value of $T_{s}$ on the total number *N* of trials per block is the robustness of interaction parameter RI, and it measures the functional requirements of success of the operation. Times ${\bar{t}}_{d}$ and ${\bar{t}}_{e}$ are the mean values for each block of the spent times for solving a conflict and executing an action. The inner speech increases times because the robot executes more steps, and the interaction with the partner involves more turns. Anyway, these times are not downtime. The tr(TRUE) value counts how many trials in each block were transparent and traceable. Obviously, the inner speech makes the trials transparent and the count is higher when the robot talks to itself (28 transparent trials in block 1 against 12 transparent trials in block 2).

The block related to the robot operation with inner speech (block 1) shows better values in terms of the number of successful trials $T_{s}$ and the consequent percentage rate RI representing the mean value of success on the total trials (0.867 of block 1 against 0.6 of block 2). The inner dialog allows solving stalemate in many cases because it enables further reasoning and interaction with the partner. Moreover, by further interaction, the robot is able to meet the partner's needs, thus increasing her/his satisfaction. When the inner dialog does not start, then the default robot's behavior does not allow the ending of task. In this case, the robot stops the execution or it alerts the partner by log messages that do not imply reasoning or interaction. The messages are just passively reproduced and the task cannot go on.

The times spent ${\bar{t}}_{d}$ and ${\bar{t}}_{e}$ are the mean values of the time parameters $t_{d_{i}}$ and $t_{e_{i}}$ computed on the total number of trials in each block (i.e. N=30). The robot spends less time when operating without inner speech. It is not surprising because the inner dialog requires more steps, which are the production of the turns. Moreover, the robot sometimes involves the partner in further interaction. The extra time the robot spends can be considered a weak point of the proposed approach, but it is not downtime. In the meanwhile, the partner assists with the robot's soliloquy or answers the robot's requests.

Finally, the transparency requirement is largely satisfied when the robot self-talks (28 transparent trials in block 1 against 12 transparent trials in block 2), as it is obvious. The partner hears the robot and knows what it wants. The cases in which the processes are considered traceable even if the robot does not talk to itself are the situations for which the corresponding tasks are simple. In these cases, no particular explanations are needed. When the tasks are complex, the transparency issue is crucial. The inner speech allows explaining them and represents a robot's fundamental skill.

## Discussion

Today, collaborative robots play a fundamental role in many contexts, ranging from industrial to domestic domains. The definitions of standards about the requirements the robots have to meet highlight the importance of the problem.

The results demonstrate the potential of robot's inner speech when it cooperates with a human. A simple cooperative task was analyzed to simulate a domestic context that needs some functional and moral requirements.

The functionality concerns the efficiency of the robot in solving the cooperative task (ISO_TS_15066, 2016). The morality regards the ethical behavior arising when the robot could infringe some social rules during the task execution. Also, it regards the transparency of the processes and the importance to make these processes traceable and reproducible (Howard and Riek, 2015). In particular, the transparency requirement is considered very important by the COMEST/Unesco (2017) standard.

By enabling a robot to talk to itself allows satisfying such requirements more times than the robot's standard operations. The robot's self-dialog provides many advantages: it makes the robot's underlying decision processes more transparent, and it makes the robot more reliable for the partner. Moreover, the interaction becomes more robust because further plans and strategies may emerge by following robot's inner speech. The robot and the partner can dialog about the situation or a conflict, and they can go out from a stalemate together.

During cooperation, several problems could cause the failure of the task. For example, the impossibility to take a specific action because the object to take is unreachable or the required movement is not feasible by the robot or again a robot's component is not working properly.

In the typical interactive session without inner speech, the robot runs the standard routines and eventually reports standard log messages. Instead, in the interactive session with inner speech, many new opportunities to face the problems can emerge. It is possible to analyze the problem and to attempt to solve it by transparently evaluating alternatives.

As shown during the threads, the partner is aware of what the robot is doing during the execution of the actions. The human is not a passive spectator of the robot's behavior because she/he can hear the explanation of that behavior.

The robot inner speech thus plays the role of a sort of “explainable” log, in a way that is meaningful for the user. The partner no longer needs to own technical knowledge to understand what happens in the robot's routines but can actively follow the robot's performance.

The robot is no longer a black box, but it is possible to look at what happens inside it and why some decisions are kept. Thus, inner speech makes the robot confidential for human.

Many other robotic contexts and functions could be investigated, thanks to such a capability. By inner speech, the robot gains a way to access its knowledge and to know its state. As previously stated, this skill is tightly linked to the self-consciousness.

Other possible functions of inner speech may be useful for robotics. Aside from the investigated cooperative scenario, inner speech may be usefully applied in robot learning or in robot regulating by overt speech or in task switching, for example, by switching attention across multiple arithmetic problems. All these aspects represent future works that can be analyzed by instilling inner speech capability in the robot. The proposed framework gives a great contribution in this scenario.

### Limitations of the study

The proposed framework for robot inner speech is a general one, and it may address many cases observed in human inner speech. However, the current robot implementation takes into consideration a simplified version of the framework.

The current grammatical structures considered in the implemented framework are limited to phrases composed by the verb, the object, and the location of the object. Many complex grammatical structures can be considered by adding different combinations of parts of speech. For the considered interactions, the proposed structures are sufficient to cover a large set of user requests.

Another limitation concerns the robot perception. Even if robot perception may include image detection and object recognition, to the purposes of the proposed framework, only the STT module is considered. The STT transformation allows decoding word sound, and it is employed to detect the user's vocal requests. An effective robot vision system would greatly enhance the capabilities of the robot. For example, inner speech may be triggered by a mirror image of the robot itself.

The current implementation of robot inner speech is based on a declarative knowledge that is fixed by the software designer: i.e., no learning or discovery of new concepts occur. However, inner speech may be an essential source of robot learning. For example, a robot, reasoning on some concepts by means of inner speech, may discover and thus may learn a new concept as a new combination of existing concepts.

### Resource availability

#### Lead contact

Further information and requests for code should be directed to and will be fulfilled by Arianna Pipitone (arianna.pipitone@unipa.it).

#### Material availability

This study did not generate new unique materials.

#### Data and code availability

The code produced for this study is available at the GitHub repository https://github.com/Arianna-Pipitone/robot-inner-speech. The repository also includes demonstrative videos of some trials.

## Methods

All methods can be found in the accompanying Transparent methods supplemental file.

## Supplemental information

Document S1. Transparent methods, Figures S1–S5, and Tables S1–S4

## Footnotes

Supplemental information can be found online at https://doi.org/10.1016/j.isci.2021.102371.
