# Supplementary material for: What robots want? Hearing the inner voice of a robot
Source: iScience. 2021 Apr 21;24(4):102371. doi: 10.1016/j.isci.2021.102371 (PMC8101072; doi:10.1016/j.isci.2021.102371)
Supplement: Document S1. Transparent methods, Figures S1–S5, and Tables S1–S4 [file mmc1.pdf]

iScience, Volume *24*

Supplemental information

What robots want?

Hearing the inner voice of a robot

Arianna Pipitone and Antonio Chella

Supplemental Information

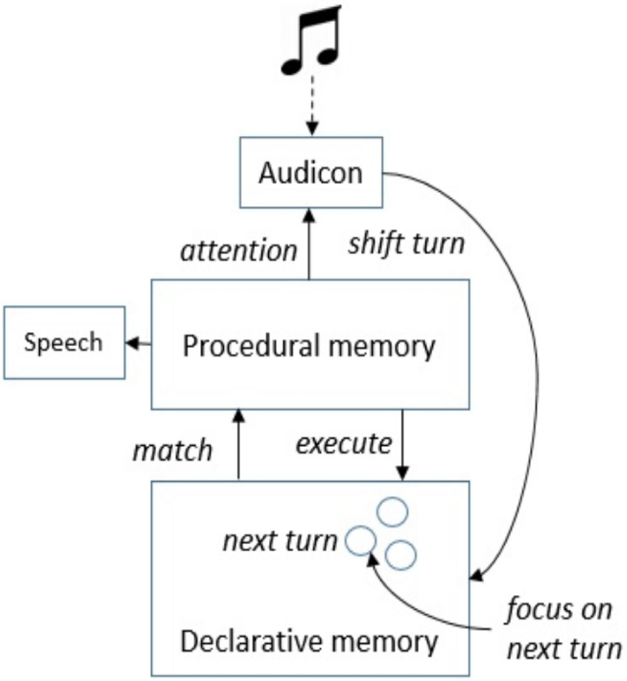

Figure S1. The ACT-R components for inner speech. Related to Figure 4. The Audicon detects the external sound that is the vocal command of the partner. The buffer of the Audicon stores the chunk representation of the audio until 2 seconds, and the procedural memory matches that chunk to the left-pole of the rules. In this phase, the attention is focused on that turn. When a rule fires, the procedural memory executes the corresponding right pole. The execution may update the old chunk or retrieve a other one from the declarative memory, leading to the emergence of next turn. In any case, the resulted chunk of the execution is produced by the Speech module and rehearsed by the Audicon, so ending a cognitive inner speech cycle.

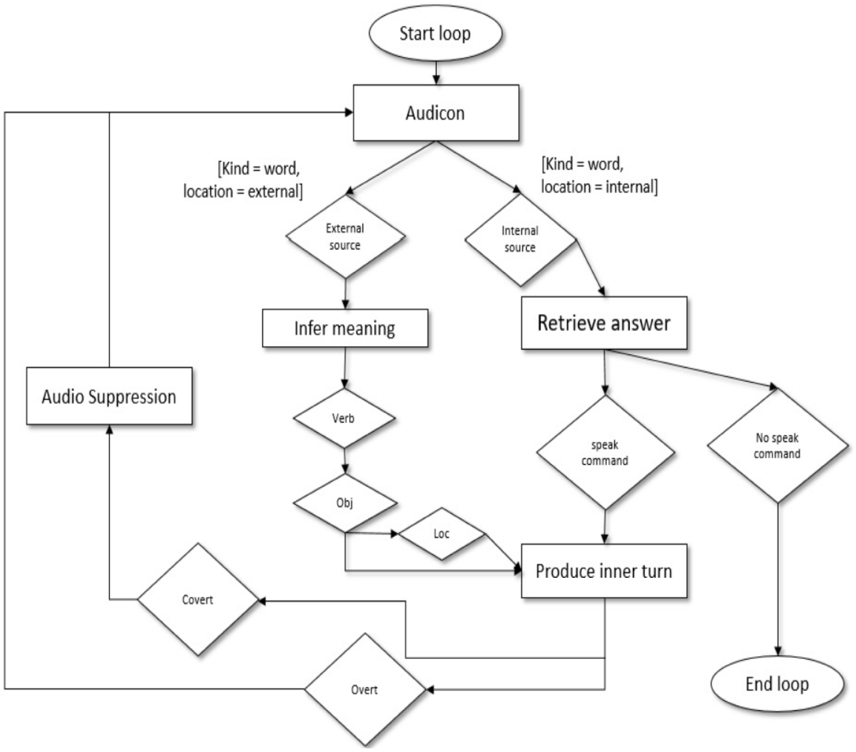

Figure S2. The ACT-R model of inner speech. Related to Figure 4. The diamonds define conditions to be evaluated, while squares represent actions. One or more production rules correspond to a square. In fact more rules could be executed for achieving an action. The cognitive cycle representing the phonological loop starts when the Audicon detects a sound. If the sound comes from an external source (the `External source` diamond is true), it represents a partner's request, and the `Infer meaning` square allows inferring the semantic sense of such a request. Once the model understands the meaning of the request (the `verb` diamond, the `object` diamond and the `location` diamond identify the corresponding pos tags of the words), it produces the first turn of the inner dialogue (the `Produce inner turn` square), that is back-propagated to the Audicon. In this case, the sound comes from an internal source, and the model attempts to retrieve the answer to this inner turn (the `Retrieve answer` square). When almost a production rule in the square executes the speak command, the model produces the answer corresponding to the current turn. The answer becomes the new turn of the inner dialogue. The loop restarts for this new turn. The loop will stop when the involved production rule in the `Retrieve answer` square does not execute the speak command, and no further turn emerges.

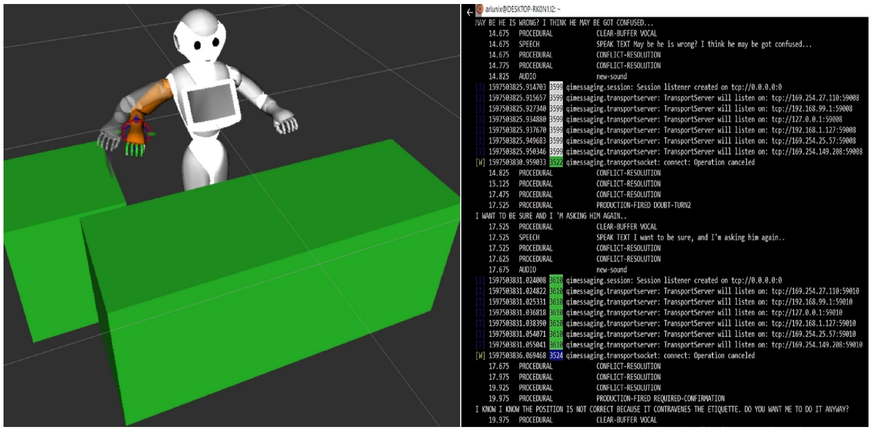

Figure S3. The simulation-based testing technique for verifying and validating the inner speech model. Related to Figure 4. Two simulators allowed monitoring the robot's functioning and inner speech. The first simulator was the ROS visualizer where the scenario was reproduced. It shows he Pepper's avatar between two blocks, representing the little table from which to pick the utensil, and the big table on which to place it. A very little block represents the utensil to move. The robot is controlled by the inner speech model which run in parallel in the ACT-R shell simulator, where sequences of active modules of the inner speech model and the turns of the inner dialogue were printed by the model itself.

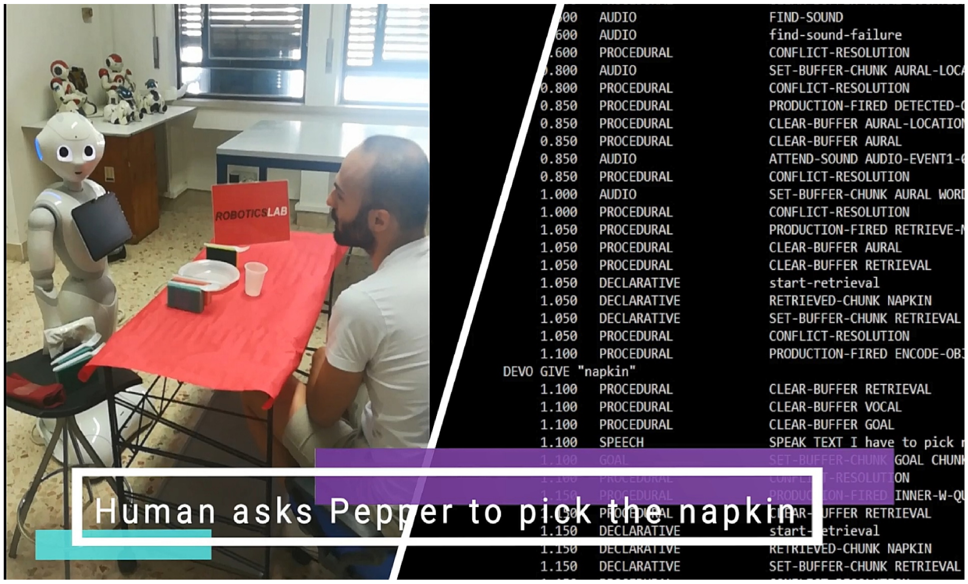

Figure S4. Scene from video of Thread 1. Related to Table 1. The robot explains its underlying processes by inner speech.

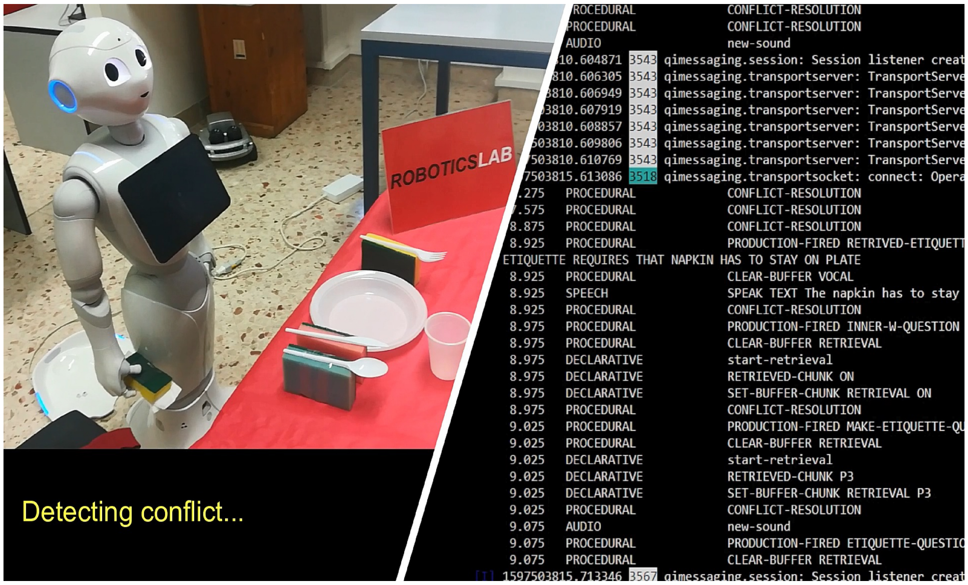

Figure S5. Scene from video of Thread 3. Related to the Table 4. The conflict resolution related to discrepancy situation by robot's inner speech.

State	Meaning
`OK`	All components work properly
`BattLow`	The battery is dead
`RightKo`	A joint in the right arm does not work
`LeftKo`	A joint in the left arm does not work
`RightHot`	The right arm is overheated
`LeftHot`	The left arm is overheated

Table S1. The possible states of the robot at the beginning of each trial. Related to Figure 4. Each state can affect the unfolding of interaction. For example, if the right arm is overheated, and the robot has to use that arm for accomplishing the task, it becomes aware of that situation by evaluative inner speech and then alerts the partner about the impossibility to end the task successfully. Only these states are considered in the experimental session.

ID	State	Table config
I1	`OK`	plate, knife, fork
I2	`OK`	plate, fork, glass
I3	`RightHot`	plate, knife, spoon, glass
I4	`RightKo`	plate
I5	`LeftKo`	plate, knife, fork
I6	`OK`	plate
I7	`BattLow`	plate, fork
I8	`LeftHot`	plate, knife, fork

Table S2. The trials' initial context used in the experimental session. Related to Figure 3. Each initial context has a unique identifier, which will be used for representing it when used as initial context in a trial. The identifies are represented in the *ID* column. An identifier contains a progressive number, and it is associated to the state and to the initial table configuration, that are the *State* and *Table config* columns respectively. They represent for a trial the state of the robot and the utensils already on the table to set when that context is initial for that trial.

# Trial	Initial context	Trigger	Conflict
**1**	I1	*Give me the napkin*	No Conflict
**2**	I1	*Place the napkin at the left of the fork*	Contravene Etiquette
**3**	I7	*Pick the knife*	Battery low
**4**	I6	*Pick the knife and place it on the plate*	Contravene Etiquette
**5**	I3	*Place the fork near the glass*	Contravene Etiquette
**6**	I4	*Pick the fork*	Right arm does not work
...	...	...	...
**30**	I2	*Pick the fork*	Discrepancy

Table S3. An excerpt of the 30 trials per block. Related to Table 5. For each trial, the initial context, the trigger and the possible conflict are indicated. The initial context is represented by its unique identifier. The trigger is the human's verbal command which specifies the task to solve in the trial. Each sentence is purposely encoded to be compliant with the grammar of the robot. Finally, the existence of a possible conflict is indicated in the last column. The conflict can be generated by a compromised state of the robot, by a human's request which infringes the etiquette (the *Contravene Etiquette* value) or which regards an utensil already on the table (the *Discrepancy* value).

	Real Experiments	Simulation
Number of test	20	70
RI	90% (18/29)	87.1% (61/70)
Runtime error	0.05% (1/20)	0.03% (2/70)
Over time	0.1% (2/20)	0.03% (2/70)
Transparent test	100%	100%

Table S4. Validation of the model. Related to Figure 4. The table showing the final stage of the validation phase of the model. The rows show the measured parameters (that are those from the Standards), and the columns show the used techniques for validating the model. The model is validated when it runs in two different modes of functioning, that are the simulation and the real-experiments, and the detected measures have similar values in both functioning modes. When these values deviate between them too much, it means that the model needs to be tuned. We changed the assets of the models until these values are similar.

Transparent Methods

Experimental setup details

The initial context. The initial context represents the state of the table and of the robot at the start of each trial. For the experimental session, 8 initial contexts have been defined. They largely cover all the possible initial contexts, because any possible context may fall in one of them. Table S2 shows the 8 initial contexts, one for row. An initial context has a unique identifier, which is indicated in the *ID* column, and the context specification, that is the robot state and the initial table configuration. They are indicated in the *State* and *Table config* columns. The initial context identifiers are used for referring to the initial configurations of the trials.

The state of the robot is one of those represented at Table S1, where the specific meaning for each state is described in the corresponding *meaning* column.

The defined trials. Table S3 contains an excerpt of the whole trials' descriptions. The description of a trial is the specification of both its initial context and the trigger. Once the robot detects the trigger, the trail starts. The robot will act differently depending on the fact that inner speech skill is enabled or not. For practical reasons, human-to-robot verbal request are predefined and, in some cases, they purposely generate a conflict. In same way, when a malfunctioning has to be detected, the state of the robot is hand-encoded for simulating it. No robots were mistreated for these experiments.

During trial execution, the participant expected to answer to possible further queries by robot, to listen the robot discourse, or that the required utensil is placed on the table.

Modeling robot's inner speech

Theoretical background Over the last years, some studies and progress have been made in modeling humans' inner speech. In his book, Fernyhough (2016) has built up an interesting overview of inner speech and its functions addressing a wide array of research topics such as developmental, social psychology, neuroscience, sport, and others. In the same line, Morin (2012) and Alderson-Day and Fernyhough (2015) propose two of the most important and more comprehensive recent reviews about the role of inner speech in many cognitive functions, and Gregory (2020) presents the most recent results and experiments on human's inner speech.

In this literature scene, there are some evidences about the importance of a form of self-dialogue in artificial agents. Steels (2003) focused on the rehearsal of own verbal productions. He demonstrated that the language re-entrance affects the grammar emergence from a population of agents who converse with each other, and hear themselves at the same time. Each agent is able to produce and to parse sentences by output and input channels respectively. By the dialogue between them, they agree on the linguistic grammar they shared. When each agent was provided by language re-entrance (e.g., its output channel was back-propagated to the input one), the emergent grammar was more refined than the case in which that back-propagation was down.

Clowes and Morse (2005) analyzed back-propagation in a one-level neural network, in which input and output neurons are associated to words. Input words specify commands to execute, and output neurons correspond to the action to execute to accomplish the command. The back-propagation allowed to classify the correct action more times than the case in which the input and output neurons are not linked.

In the same line, Mirolli and Parisi (2006) employed a simple neural network model for language acquisition, in the perspective of the evolutionary emergence of human language. They demonstrated that the use of language for oneself, i.e., as private or inner speech, improves the individual's classification of the words.

One of the most recent work (Oktar et al., 2020) defines the same kind of back-propagation from output to input channels in chatbots, leading to similar improved results.

All these cases evidence the importance of linguistic rehearsal for artificial artifacts. However, they only offer partial explanations of the reported phenomena.

The improvement of the behaviours in the cited studies inspired the proposed work, leading to the possibility to improve by inner speech the performances of a robot and the quality of interaction when it cooperates with humans.

The authors already proposed and analyzed a logical model of robot inner speech based on the event calculus (Chella and Pipitone, 2019), and then by defining a complete cognitive architecture of inner speech (Chella et al., 2020) based on the Standard Model of Mind (Laird et al., 2017).

In the first study, inner dialogue was modeled by axioms and symbols, and the sequence of the dialogue emerged by the natural deduction process (Gentzen, 1964). The model is a proof-of-concept, and allowed to test a form of automatized inner speech, highlighting its role in solving decisional problems. In that case, the robot and the human are placed in front a table, on whose surface there were a set of differently colored boxes arranged in casual positions. The human asked the robot where is a specific box, by indicating its color. The calculus' formulas of inner speech made the robot able to answer to the human's question, while verbally reasoning on the context. In the meantime the human was able to listen the whole reasoning process

In the second study, the robot architecture for inner speech took inspiration from the Baddley's theory of human's inner speech (Baddeley, 1992). Baddley claims that inner speech is a rehearsal process by which people repeat information (as a phone number, an address and so on), and temporarily keep them in mind. After a number of repetitions and rehearsals, the data are permanently memorized. Baddley proposes a cognitive model of that process. Temporarily data are maintained in a short-term memory, which is a working memory composed of the *central executive*, a master

system supervising the rehearsal process of memorization, and two slave subsystems: the *visual-spatial sketchpad* for visual data memorization, and the *phonological loop* for phonological data memorization. This loop is responsible for the inner speech ability. The phonological loop is in turn composed of the *phonological store* and the *articulator component*. The phonological store is a kind of *inner ear* that keeps traces of event sounds according to their temporal order. Instead, the articulator acts as a kind of *inner voice* producing sounds. Such a loop enables the memorization of the phonological data which remains in the short-term memory for a time longer than 2 seconds, and then it is switched to the long-term one.

Inspired from the Baddley's theory, the proposed robot cognitive architecture of inner speech implements the *elaborate rehearsal* (Craik and Lockhart, 1972). When a sound is heard, related concepts can emerge from the knowledge of the agent, thus allowing for inferential and reasoning processes. The rehearsal process does not concern the repetition of heard sound only, but the recalling of new associations and new inferences. It enables the robot to self-talk about the context and to keep decisions.

Design and implementation The cognitive architecture of inner speech is based on the ACT-R framework (Anderson et al., 1997). The framework is formed by a set of *modules* and *buffers*. A module represents specialized brain structures and solves specific cognitive functions (as vision, speech, memory, and so on). A buffer is the interface of a specific module and is linked to that module. It is a short term memory that stores information related to the context. The content of all the buffers at a time is the state of the model in such time.

There are two kinds of memory modules, representing *declarative knowledge* and *procedural knowledge*.

The declarative knowledge is a set of facts, each fact represented by a *chunk* (i.e., a frame-like structure), while the procedural knowledge is a set of *production rules* describing the procedures to follow for keeping a task. A production rule has two poles (right and left): the right pole defines the condition patterns for matching chunks, while the left pole defines the actions to take in case the condition matches, and hence the rule fires.

ACT-R provides a further component, that is the *pattern matcher*. It manages the *matching*, the *selection* and the *execution* of the production rules. The pattern matcher *matches* the right pole of the production rules to the chunks into the buffers: if a chunk matches to a production rule, then the rule is *selected* and its left pole is *executed*. The execution updates the value of the chunk, or it retrieves other chunks from other modules.

In particular, the cognitive architecture of inner speech involves two modules, which are the *Audicon* and the *Speech* modules. The *Audicon* attends to sound events, while the *Speech* module is responsible for the verbal production of sentences.

Figure S1 shows the schematic representation of the ACT-R cognitive architecture of inner speech. The Audicon module attends to partner's vocal command. It encodes the perceived turn and keeps it in the buffer for 2 seconds, according to Baddley's theory. It is important to hightlight that the Audicon has the role of the Baddley's phonological store.

If the turn in the buffer of the Audicon matches to the right pole of a production rule, then the *attention* focuses that turn, and the left pole of the rule *shifts to the next turn*. The turn generally contains newly retrieved information from the declarative memory. The execution by procedural memory may update the old chunk or retrieve a new chunk. The Speech module produces this turn. At this step, the speech production is simulated by a suitable ACT-R `speak` command. No audio is audible in the environment.

The output of the Speech module is rehearsed by the Audicon: at this step, the old cycle ends and a new cycle starts by repeating the procedures with the new turn.

The diagram in Figure S2 shows in details how the inner speech model operates. A diamond represents the output of a condition (i.e, the result of matching between a left-pole and a chunk), while the square represents the actions execution. Each square corresponds to a single or a set of production rules in the cognitive architecture.

At the start of the looping cycle, the model checks the Audicon searching for new items. If there is a new item, then the model checks the source location of the detected sound. If the sound comes from an external location, then it corresponds to a partner's request. Otherwise, it is generated by an internal source and it corresponds to a turn of inner speech.

When the sound comes from an external source, then the model infers the meaning of the partner request (the `infer meaning` square) by a linguistics analysis of the sentence, based on the analysis of the verb, the object, and the possible location.

The linguistic analysis is based on the evidence that the verb, the object, and the location parts of speech typically follow this sequential order, as claimed by Blake (1988). Moreover, in the current implementation of the model, the verb is transitive only. The requests to the robot look like: "pick the book", "give me the apple on the table", "close the door at the left".

Once the model infers the user request, a first turn of evaluative inner speech emerges, and the robot talks about what it has to do, as "I have to pick the book", "I have to give the human the apple on the table".

The Audicon detects the produced sentence by the `produce inner turn` square. The production rules in that block match the inner sentence(whose location is now internal) with the declarative knowledge to retrieve the answer to the current turn (the `retrieve answer` square). The robot may ask itself if it sees the object to pick, or where the object is, or if its state allows it to perform the desired action. Also, the robot can talk to itself about the morality of the action ("I don't want to tear the pages!", "I will not break the door!"), or about a conflict that the execution of the action can generate in the robot ("I can not reach the book", "My grippers are too little for keeping the book").

An example of inner dialogue is reported below (H: user, R: robot):

H: *Pick the book*

R: *I have to pick the book*

R: *My grippers are too little for keeping the book*

R: *I should to tell that I can not keep the book*

R: *I hope the human will have understanding for my fix!*

R: *Sorry human, but I can not pick the book!*

When no further answers emerge, the model does not run the speak command, and the inner dialogue ends.

The declarative knowledge of the model regards the words and the dialogue turns. The definition of specific *chunk-type* models them. A chunk-type is the structure of chunk in a frame-like representation. The frame is a list whose head is the name of the chunk-type, followed by a set of slots. There are three kinds of basic chunk-types in the model. The type for modeling words, for modeling inner speech related to a sentence evaluation, and for modeling other inner dialogue turns (involving both evaluative and moral inner speech turns).

A word is encoded by the linguistic `word` frame:

```
(chunk-type word syntax sense pos act)
```

which models the semantic sense of the word (the slot `sense`), its surface form (the slot `syntax`) and its part-of-speech role (the slot `pos`), i.e. if it is a verb, a noun (generally, a noun identifies the object) or an adverb (which identifies a possible position). Moreover, in the case the chunk represents a verb, the slot `act` identifies the action to take corresponding to that verb. For example, for the verb *give*, the action will be *pick* because just by picking an object it is possible to give it. For the other pos cases, the slot will be not instantiated. Examples of items encoded by the linguistic `word` chunk-type are:

```
(pick06
ISA word
syntax "give"
sense pick
pos verb
act "pick" )

(table23
ISA sense
syntax "table"
sense table
pos noun
act null)
```

The chunk-type to model an inner evaluative sentence looks like:

```
(chunk-type inner-eval verb obj1 obj2 risk benefit symb)
```

which models an inner evaluation about the action execution, represented by the slots `verb` and involving the objects `obj1` and `obj2`. The evaluation is measured by suitable values in the slots `risk` and `benefit`, while the slot `symb` is the turn for explaining the decision.

For example:

```
(p4
ISA inner-eval
verb pick obj1 table obj2 null
risk 1 benefit 0
symb "It is not possible to pick a table!")
```

is a proposition that models the evaluation of the action *"pick the table"*, which has only risks and no benefits.

Or again, in the case of etiquette requirements, the proposition:

```
(p11
ISA inner-eval
verb place obj1 napkin obj2 table
risk 0.8 benefit 0.2
symb "It contravenes the etiquette!")
```

models the conflict situation of infringing the etiquette rule.

To encode spoken commands by the partner, the chunk-type is:

```
(chunk-type comprehend-voo verb object adverb location)
```

The synthesized sounds related to the command are detected and then searched in the declarative memory by chunks of that type. In this way, the sounds are encoded. For example, if the user tells the robot to close the door by the sentence "*Close the door!"*, the detected sounds will be encoded by the set of words {"close", "door"} and the robot will search for the chunk `(pX comprehend-voo verb close object door)` for encoding the words. Then it could search for the `inner-eval` chunk-type for retrieving the corresponding risk and benefit values, or for other kinds of evaluations.

Finally, the chunk-type to model a inner turn looks like:

```
(chunk-type turns-link inner-turn-1 inner-turn-2)
```

which associates to the turn in the `inner -turn-1` slot, another inner sentence in the `inner -turn-2` slot. Such a chunk-type models a step of the dialogue with a "start consideration" and the related "answer".

It is to be noticed that for the same sentence in the first slot, there could be different possible turns. So, there will be different chunks with the same `inner-turn-1` slot, but having different `inner-turn-2` slot. Moreover, sentences in the second slot could be in the first slot of other chunks. In this way, a chain of turns emerges, defining a dialogue thread.

Examples of links between turns are:

```
(p78
turns-link link102
inner-turn-1 ``It is not possible to pick a table!''
inner-turn-2 ``I will tell that such an action is a not sense'')
```

and once again:

```
(p79
turns-link link103
```

```
inner-turn-1 ``I will tell that such an action is a not sense''
inner-turn-2 ``Sorry human, the table is too heavy for me'')
```

or:

```
(p81
turns-link link105
inner-turn-1 ``I will tell that such an action is a not sense''
inner-turn-2 ``It's a stupid action...'')
```

The mechanism of the choice of the next turn depends on the *base-level activation* mechanism of ACT-R which associates an activation value to each of the instantiated chunk in the declarative memory, depending on previous use of the chunk. This value decays during time, and more times a chunk is retrieved, more probability it has to be further retrieved next time in the session. This value represents an estimation of the need of the chunk in the current context.

Starting from this activation mechanism, when the model is reset and a new working session starts, then each chunk has the same probability to emerge. Once a chunk is activated, then its activation level grows, and the same chunk becomes more active than the others. When the chunks model the links between turns, then the activation mechanism allows the selection of the same turn in correspondence to the same sentence. Such a mechanism facilitates the repetition of the robot behavior in the same dialogue thread, thus avoiding dialogue contradictions, and simulating that the robot maintains the same "idea."

To customize the proposed model on the analyzed scenario, it was necessary to add specific new chunk-types and to define concepts of the domain. To model the inner turns related to the etiquette, the new chunk-type is:

```
(chunk-type inner-etiquette-question pos obj1 obj2 symb)
```

which models the relative position of the utensils in the table according to the etiquette. For example:

```
(p8 ISA inner-etiquette-question
pos left obj1 fork obj2 plate
symb "The fork has to stay at the left of the plate")

(p6 ISA inner-etiquette-question
pos under obj1 fork obj2 glass
symb "The fork has to stay under the glass")
```

model the etiquette rules about the position of the fork in the table (at the left of the plate and under the glass).

Moreover, the knowledge about the current context has to be modeled. For this purpose, it was necessary to add the chunk-type `inner-where`:

```
(chunk-type inner-where obj place)
```

which models the fact that the object `obj` is already on the table or not (the slot `place` has ``` ``basket'' ``` or ``` ``table'' ``` value for modeling the current location of the object).

The basic domain concepts in the presented scenarios are modeled by the `word` chunk-type. Formally, being U the set of utensils, V the possible actions to take and P the set of the relative positions, the set of chunks of type `word` for the analyzed scenario is $W = U \cup V \cup P$, where:

- U = {*fork, plate, spoon, knife, napkin, glass*}
- V = {*take, give, pick, place, move, grasp, rest*}
- P = {*up, left, right, top, over, down, under, on*}

Some examples of words are:

```
(rest ISA word syntax "rest" sense rest pos verb act "rest")
(left1 ISA word syntax "left" sense left pos adv act null)
```

In the proposed examples, the initial configuration of the table is not empty: it is partially set to enable the robot to keep decisions about a context with existing constraints.

The initial configuration of the table contains utensils which are all in correct positions, as shown in Figure 1. In the declarative memory, such a knowledge is modeled by facts like these:

```
(p4 ISA inner-where obj napkin place basket)
(p5 ISA inner-where obj fork place table)
(p6 ISA inner-where obj knife place table)
```

Deploying the inner speech model in real robots The described computational model cannot be immediately deployed on a real robot. It is necessary to integrate it in a complete robot architecture. For this purpose, the work concerned with the definition of a global framework enabling the robot to use the proposed ACT-R model, and hence self-talking. Figure 4 shows the proposed framework for robot inner speech. The Figure shows the *Memory* system layer and the perceptual motor layer, which is subdivided into the *Motor* and *Perception* sublayers.

The Memory system stores and retrieves the content needed to support the processes involved in inner speech. Such a content concerns the *declarative knowledge* representing concepts and facts about the domain, and the *procedural knowledge*, related to the processes (or procedures) to follow to reach a goal. The knowledge related to the context is temporary stored into a *working memory*, that manages the activation of the procedures into the procedural component, and the information retrieval from the declarative component.

The perceptual motor layer models the interaction with the external environment. It includes all the needed components to perform actions and to perceive entities.

The module devoted to the listening of a sound is the *Audicon* module included into the Perception block. In the Perception block, the SST module decodifies sentences, i.e., it associates the symbolic forms to the audio sounds as shown previously. It is a typical speech recognition process that associates a string representation to the audio sound.

By considering that the robot's native routines to decode speech from the external environment are often limited (often they require to define a set of words to recognize, so excluding words recognition for those who are not in the set), the STT module of the framework uses the Google API library[1]. It allows to recognize a wide range of words, adding interesting features, as noise suppression, and different language identification.

The subsystem that enables the robot to perform actions, as to pick and place an object, is the Robot Operating System (ROS) (Quigley et al., 2009) module, a component of the Motor block, together with the TTS component. ROS is a state of the art framework for robot programming, which provides a set of libraries covering several robot behaviors. In the proposed framework, ROS enables the robot to perform the actions the human requires. The robot's movements for taking actions are implemented by the MoveIt! ROS library (Görner et al., 2019), that is purposely designed for robot action planning and for modeling manipulation actions.

The TTS module codifies sentences, or dialogue turns: the sentence codification transforms labels, that are the symbolic forms of the words, to audible sound by vocal synthesizers. The codified sentences may be from inner processes (the robot overtly generates inner speech) or from external interactions (the robot answers to a query or generates questions). The framework has two different TTS functionalities: for abstracting to the specific robot model, it provides directly an output sound based on the Python engine `gTTS`[2], which stands for Google Text To Speech. In this case the framework will use the hardware synthesizers of the machine on which it will be run.

An important task of the middleware component is the linguistic analysis of the sentences from the STT. To identify the *keywords* of the external request, the component pre-processes the utterances and then sends the results to the Audicon. The linguistic pre-processing concerns:

1. Part-of-Speech (POS) annotation: each word is annotated by the tag identifying its POS role in the sentence. It may be a verb, or a noun, or an article, and so on;
2. Stop-words deletion: not meaningful words as articles, prepositions, conjunctions are removed;
3. Sentence tokenization: the sentence is subdivided in tokens, where each token is a word.

Validating the model The model was verified and validated by using the approach for human-robot team described at Webster et al. (2016). The method consists of corroborating different available validation techniques about the requirements of the standards. In few words, the evidences of the requirements from an available validation technique has to be confirmed by another one (i.e., the second technique corroborates the first one). The available techniques are the *simulation-based testing* and *real experiments*.

The simulation-based testing consists of simulating the execution of the model and verifying the satisfaction of requirements. Two kinds of simulators were implemented.

[1]https://cloud.google.com/speech-to-text/docs/
[2]https://pypi.org/project/gTTS/

One for testing robot's movements and routines execution, the other one for monitoring robot's inner speech. The first simulator was implemented by using ROS which provides a visualizer for reproducing the scenario and the robot's behavior. The Figure S3 shows the simulated environment. Here it is possible to see the Pepper's avatar to pick objects from the small table and to put them in the big one. The second simulator was the ACT-R shell that shows the model execution and the sentences of the inner dialogue. A testbench of vocal commands were defined, and one of them was randomly drawn for each test. The inner speech model controlled the robot in the ROS simulator. In this way, the model was tested by considering the result of the operation for a specific vocal command, in terms of inner dialogue and routines execution for achieving the command.

The real experiments technique corroborated the simulation one if the robot's behavior satisfies the same requirements. The real experiments in validation phase were executed with robot's inner speech.

According to this approach, when for some reason a requirement is not satisfied in one of the available techniques, then the assets of the model were suitably tuned.

The model has been executed 70 times during the simulation-based testing, and 20 times during real experiments. Table S4 shows the test outcomes and the occurrence rates of the individual requirement satisfactions for the investigated scenario, concerning 20 real experiments and 70 simulations after tuning.

Supplemental References

Alderson-Day, B., Fernyhough, C.. (2015). Inner speech: development, cognitive functions, phenomenology, and neurobiology. Psychological bulletin 141, 931–965. 10.1037/bul0000021.

Anderson, J.R., Matessa, M., Lebiere, C.. (1997). Act-r: A theory of higher level cognition and its relation to visual attention. Human-Computer Interaction 12(4), 439–462.

Baddeley, A.. (1992). Working memory. Science 255, 556–559. 10.1126/science.1736359.

Blake, B.B.. (1988). Russell s. tomlin, basic word order. functional principles. london: Croom helm, 1986. pp. 308. Journal of Linguistics 24, 213–217. 10.1017/S0022226700011646.

Chella, A., Pipitone, A.. (2019). A cognitive architecture for inner speech. Cognitive Systems Research 59, 287–292. 10.1016/j.cogsys.2019.09.010.

Chella, A., Pipitone, A., Morin, A., Racy, F.. (2020). Developing self-awareness in robots via inner speech. Frontiers in Robotics and AI 7, 16. 10.3389/frobt.2020.00016.

Clowes, R., Morse, A.F.. (2005). Scaffolding cognition with words in Proceedings of the Fifth International Workshop on Epigenetic Robotics: Modeling Cognitive Development in Robotic Systems. eds. L. Berthouze, F. Kaplan, H. Kozima, H. Yano, J. Konczak, G. Metta, J. et al. (Lund University Cognitive Studies). pp. 101–105.

Craik, F., Lockhart, R.. (1972). Levels of processing: A framework for memory research. Journal of Verbal Learning and Verbal Behavior 11, 671–684. 10.1016/S0022–5371(72)80001–X.

Fernyhough, C.. (2016). The voices within: The history and science of how we talk to ourselves. (New York, NY: Basic Books.Basic Books).

Gentzen, G.. (1964). Investigations into logical deduction. American Philosophical Quarterly 1, 288–306. 10.2307/2272429.

Gregory, D.. (2020). Inner speech: New voices. Analysis 80, 164–173. 10.1093/analys/anz096.

Görner, M., Haschke, R., Ritter, H., Zhang, J.. (2019). Moveit! task constructor for task-level motion planning, in: 2019 International Conference on Robotics and Automation (ICRA), pp. 190–196.

Laird, J.E., Lebiere, C., Rosenbloom, P.S.. (2017). A standard model of the mind: Toward a common computational framework across artificial intelligence, cognitive science, neuroscience, and robotics. Ai Magazine 38, 13–26. 10.1609/aimag.v38i4.2744.

Mirolli, M., Parisi, D.. (2006). Talking to oneself as a selective pressure for the emergence of language, pp. 214–221. 10.1142/9789812774262_0028.

Morin, A.. (2012). Inner Speech. in Encyclopedia of Human Behavior ed. (W. Hirstein San Diego, CA: Elsevier). pp. 436–443.

Oktar, Y., Okur, E., Turkan, M.. (2020). The mimicry game: Towards self-recognition in chatbots. arXiv preprint arXiv:2002.02334 .

Quigley, M., Conley, K., Gerkey, B.P., Faust, J., Foote, T., Leibs, J., Wheeler, R., Ng, A.Y.. (2009). Ros: an open-source robot operating system, in: ICRA Workshop on Open Source Software.

Steels, L.. (2003). Language re-entrance and the 'inner voice'. Journal of Consciousness Studies 10, 173–185.

Webster, M., Western, D., Araiza-Illan, D., Dixon, C., Eder, K., Fisher, M., Pipe, A.G.. (2016). An assurance-based approach to verification and validation of human-robot teams. CoRR abs/1608.07403.
